# The Relationship between Frequently Used Glucose-Lowering Agents and Gut Microbiota in Type 2 Diabetes Mellitus

**DOI:** 10.1155/2018/1890978

**Published:** 2018-05-07

**Authors:** You Lv, Xue Zhao, Weiying Guo, Ying Gao, Shuo Yang, Zhuo Li, Guixia Wang

**Affiliations:** Department of Endocrinology and Metabolism, The First Hospital of Jilin University, Changchun, Jilin, China

## Abstract

Metabolic diseases, especially diabetes mellitus, have become global health issues. The etiology of diabetes mellitus can be attributed to genetic and/or environmental factors. Current evidence suggests the association of gut microbiota with metabolic diseases. However, the effects of glucose-lowering agents on gut microbiota are poorly understood. Several studies revealed that these agents affect the composition and diversity of gut microbiota and consequently improve glucose metabolism and energy balance. Possible underlying mechanisms include affecting gene expression, lowering levels of inflammatory cytokines, and regulating the production of short-chain fatty acids. In addition, gut microbiota may alleviate adverse effects caused by glucose-lowering agents, and this can be especially beneficial in diabetic patients who experience severe gastrointestinal side effects and have to discontinue these agents. In conclusion, gut microbiota may provide a novel viewpoint for the treatment of patients with diabetes mellitus.

## 1. Introduction

Over the past few decades, metabolic diseases such as type 2 diabetes mellitus (T2DM), obesity, dyslipidemia, and cardiovascular diseases have become major public health issues all over the world. Accordingly, an increasing amount of research has been conducted to further investigate the pathogenesis, phenotypes, and treatments of such diseases. One of the most common metabolic disorders is T2DM, which is characterized by chronic hyperglycemia that can be attributed to genetic and/or environmental factors. Recently, the role played by gut microbiota in T2DM has gained increasing attention, with several studies investigating the composition and function of gut microbiota in T2DM [1].

It was found that, for a standard-weight male, the ratio of bacterial cells to human cells is approximately 1 : 3, with an uncertainty of 25% and a variation of 53% [2]. Several studies reported an association between gut microbiota and metabolic diseases [3–9], with some studies presenting the differences between gut microbiota in T2DM patients and healthy individuals [3, 10]. For example, Qin et al. [11] reported that many opportunistic pathogens, such as *Bacteroides caccae*, *Clostridium hathewayi*, *Clostridium ramosum*, *Clostridium symbiosum*, *Eggerthella lenta*, and *Escherichia coli*, are enriched in T2DM patients. In addition, Karlsson et al. [12] reported an increase in *Lactobacillus* spp. (*Lactobacillus gasseri* JV-V03, *Lactobacillus gasser*i SJ-9E-US, *Lactobacillus gasseri* 202-4, and *Lactobacillus salivarius* ACS-116-V-Col5a) and a decrease in *Clostridium* spp. (*Clostridium beijerinckii* NCIMB 8052, *Clostridium* sp. 7_2_43FAA, *Clostridium botulinum* B str. Eklund 17B, *Clostridium botulinum* E3 str. Alaska E43, and *Clostridium thermocellum* DSM 1313) in T2DM patients. Treatment with metformin affects gut microbiota, and thus, it might be an important confounder in the above studies. Gut microbiota were found to control body weight after bariatric surgery, regulate plasma glucose and insulin levels, maintain the intestinal epithelial barrier integrity, and lower the levels of inflammatory cytokines [13–16]. Moreover, some probiotic supplements exhibit beneficial metabolic properties [15–17]. All this information suggests that gut microbiota may be involved in the etiology of diabetes mellitus.

Short-chain fatty acids (SCFAs) including acetate (C2), propionate (C3), butyrate (C4), and valerate (C5) are produced by anaerobic bacteria through the fermentation of nondigestible dietary polysaccharides in the colon [18–20]. Some researchers suggested that a common form of gut microbiota alteration in T2DM is the reduction in butyrate-producing bacteria [11, 21]. Sodium butyrate was found to lower plasma glucose and lipid levels, improve insulin resistance, and reduce gluconeogenesis in diabetic rats [22]. Thus, SCFAs might have a promising role in the prevention and treatment of diabetes mellitus.

Currently available therapeutic options for T2DM, especially glucose-lowering agents, target different pathophysiologic processes. A large body of evidence suggests that gut microbiota and SCFAs exhibit positive effects on glucose-lowering agents in T2DM. Glucose-lowering agents can influence the composition of gut microbiota [23, 24] and affect the production of SCFAs, thereby leading to significant beneficial effects [1, 25, 26]. Moreover, adverse effects of glucose-lowering agents may be improved by altered gut microbiota [27, 28]. This review summarizes current information on the relationship between frequently used glucose-lowering agents and gut microbiota (Table 1) to better understand the role of the intestinal microenvironment in the treatment of diabetes mellitus.

## 2. Metformin and Gut Microbiota

### 2.1. Metformin Mechanism of Action and Side Effects

Metformin is a biguanide that reduces hepatic glucose production, increases glucose uptake by peripheral tissues, and activates AMP-dependent protein kinase (AMPK). Metformin is actively transported into cells by organic cation transporters (OCT) [29, 30]. The mechanism of action of metformin is not yet fully understood. In an experimental study on mice with alanine knock-in mutations in both acetyl-CoA carboxylase (Acc) 1 (Ser79) and Acc2 (Ser212), treatment with metformin reduced hepatic lipogenesis and lipid accumulation via activating AMPK and inhibited both Acc1 and Acc2, thereby increasing insulin sensitivity [31]. Duca et al. [32] found that metformin could activate duodenal mucosal AMPK and attenuate hepatic glucose production in a rat model of high-fat diet (HFD). In addition, Miller et al. [33] found that metformin lowers the fasting glucose level by inhibiting glucagon-stimulated cyclic adenosine monophosphate production, which leads to reduced protein kinase A activity and glucagon-stimulated glucose output. Metformin was also found to alter intestinal microbiota [34].

Currently, metformin is the first-line treatment for T2DM and is recommended by the American Diabetes Association and the European Association for the Study of Diabetes with proven efficacy, safety, and low cost [35]. It can lower plasma glucose and insulin levels, improve lipid profiles, and promote modest weight loss. An extended-release formulation of metformin that has fewer gastrointestinal (GI) side effects, for example, diarrhea, anorexia, nausea, and metallic taste, is also available [21, 29]. About 30% of the patients treated with metformin report suffering from GI adverse effects [1]. In a previous clinical study in which 360 T2DM patients were administered a new prescription of metformin for 3 months, about 88% of the participants reported single or multiple GI symptoms, including diarrhea, heartburn, and nausea [36]. Possible underlying mechanisms for metformin adverse effects include stimulation of intestinal serotonin secretion, changes in incretin and glucose metabolism, bile salt malabsorption, and high intestinal metformin concentrations after oral administration [37]. Since OCT1 is involved in the absorption of metformin from the intestinal lumen, its inhibition results in increased metformin intolerance [37]. Recent data illustrated that delayed-release metformin performs its action predominantly in the intestine, so it is considered to be safe for T2DM patients with renal impairment [38].

### 2.2. The Effect of Metformin on Gut Microbiota in T2DM

Metformin has pleiotropic effects. It accumulates mainly in the intestine, where its concentration is nearly 300 times higher than its plasma concentration [39]. Thus, the intestine is the major site of metformin exposure and is responsible for its glucose-lowering effect [38]. In 1984, Bonora et al. [40] found that intravenous administration of metformin did not improve glucose metabolism compared to oral administration, which suggests the importance of the intestine in the regulation of glucose metabolism by metformin. Metformin was found to increase the life span of *Caenorhabditis elegans* cocultured with *Escherichia coli* by altering microbial folate and methionine metabolism [41], which suggests that the effects of metformin on aging in nematodes are microbiota dependent. Recently, the relationship between metformin and the gut has been comprehensively reviewed [21]; researchers indicated that metformin can affect the gut microenvironment by modulating glucose uptake and utilization, increasing glucagon-like peptide-1 (GLP-1) and bile acid levels and altering gut microbiota. In conclusion, the interaction between metformin and gut microbiota may contribute to the pleiotropic effects of metformin.

A large body of evidence confirms the effects of metformin on intestinal microbiota. The abundance of *Akkermansia muciniphila* was found to be decreased in obesity and diabetes [9], while higher baseline levels were associated with improvements in the cardiometabolic parameters of obesity [11, 42]. Experimental studies revealed that the abundances of *Akkermansia muciniphila* [43, 44] and *Clostridium cocleatum* [43] increased significantly in HFD-fed mice after metformin treatment. The higher abundance of *Akkermansia muciniphila* was involved in maintaining mucin layer integrity [45, 46], and the changes in gut microbiota were attributed to SCFA-producing bacteria in human and animal guts [45, 46]. It is suggested that the effects of metformin on the abundance of these species may indirectly contribute to its modulatory effects on glucose metabolism and other metabolic processes. These results suggest that the therapeutic effect of metformin might be partially mediated by the intestinal tract.

Forslund et al. [1] found that metformin-treated T2DM patients exhibited decreased abundances of *Intestinibacter* spp. and increased abundances of *Escherichia* spp. However, the latter was only found in Danish and Swedish populations. In a cross-sectional study that recruited 145 European old women with T2DM, impaired glucose tolerance, or normal glucose tolerance, patients treated with metformin showed different gut microbial composition than those who were not treated with metformin [12]. Napolitano et al. also evaluated T2DM with and without metformin monotherapy to characterize the gut-based mechanisms of metformin, and their results showed that *Adlercreutzia* spp. were significantly elevated in fecal samples of metformin-treated patients [30]. Wu et al. [24] found that the altered microbiota mainly belonged to the phyla *γ*-Proteobacteria and Firmicutes. They found an increase in *Escherichia* spp. and a decrease in *Intestinibacter* spp., as well as a significant increase in fecal propionate and butyrate concentrations in the metformin group. A possible underlying mechanism for metformin-microbiota interaction is regulating the expression of genes encoding for metalloproteins in gut bacteria. These results demonstrate the influence of metformin on gut microbial diversity and the role of the intestine in the glucose-lowering effect of metformin.

Unfortunately, alteration of gut microbiota by metformin may contribute to its GI intolerance [21]. Greenway et al. [27] reported a case in which a 30-year-old man newly diagnosed with T2DM was treated with a cobiotic supplement containing inulin, beta-glucan, and blueberry pomace extract for 8 weeks. This patient exhibited balanced glycemic control and alleviated GI side effects of metformin. In addition, Burton et al. [28] found that the combination of metformin with GI microbiome modulator (GIMM) in T2DM patients who experienced GI intolerance to metformin resulted in better glucose tolerance compared to placebo and significantly improved fasting glucose levels. These results demonstrate that a safe dietary supplement may improve the efficacy and tolerability of metformin, possibly through the alteration of gut microbiota. Thus, gut microbiota may serve as a novel approach to alleviate metformin adverse effects and consequently improve patient compliance.

## 3. The Effect of Acarbose on Gut Microbiota in T2DM

Accumulating evidence suggests that postprandial blood glucose level is a powerful predictor of diabetic cardiovascular events [47, 48]. As a classical *α*-glucosidase inhibitor, acarbose lowers postprandial blood glucose levels by delaying glucose absorption, as it inhibits the enzyme that cleaves oligosaccharides into mono- and disaccharides in the intestinal lumen [29]. The control of postprandial hyperglycemia is especially important in Asia due to the traditional carbohydrate-rich dietary pattern [49]. In a randomized, open-label, noninferiority clinical trial recruiting Chinese patients newly diagnosed with T2DM, treatment with acarbose as initial therapy showed similar glucose-lowering efficacy to that of metformin [50]. Therefore, acarbose is an effective and safe antidiabetic agent, especially for the control of postprandial hyperglycemia. The major cause of acarbose side effects (e.g., diarrhea, flatulence, and abdominal distention) is the increased delivery of oligosaccharides to the large intestine. However, these side effects can be ameliorated by gradual upward dose titration [29]. Previous studies indicated that acarbose treatment could reduce the risk of cardiovascular events in diabetic patients [51–54]. The detailed underlying mechanisms for this cardiovascular protective function are only partially understood [55], but they can be attributed to the ability of acarbose to neutralize oxidative stress by increasing H_2_ production in the GI tract (GIT) [56]. However, the results of Chang et al. did not support a cardiovascular protective effect of acarbose compared to metformin in Taiwan populations [57].

A series of recent clinical trials clarified the relationship between acarbose and gut microbiota. For example, in a clinical study, 95 Chinese T2DM patients were distributed into 2 groups; one group was treated with acarbose while the other was not. At baseline, diabetic patients showed lower levels of *Bifidobacterium longum* and higher levels of *Enterococcus faecalis* than healthy volunteers [55]. After 4 weeks of treatment, acarbose-treated patients showed increased *Bifidobacterium longum* and decreased lipopolysaccharide and prothrombin activator inhibitor-1 levels [55]. These results suggest that acarbose treatment could alter gut microbiota and reduce the levels of inflammatory cytokines in diabetic patients. Another randomized, double-blind, controlled crossover trial recruiting prediabetic Chinese individuals demonstrated that acarbose treatment significantly altered the diversity and composition of gut microbiota [58]. In addition, Gu et al. [23] revealed that acarbose could regulate the gut microbiota of T2DM patients, thereby modulating bile acid metabolism and contributing to beneficial effects on host metabolism.

Although *α*-glucosidase inhibitors have been widely used clinically, there is an ongoing interest in probiotics or gut microbiota as biotherapeutic agents for some metabolic diseases. Panwar et al. [59] found that *Lactobacillus* strains exhibit glucosidase-inhibitory effects and regulate blood glucose responses to carbohydrates *in vivo*. As mentioned previously, SCFAs play an important role in diabetes mellitus. Acarbose was found to increase serum butyrate levels in individuals with impaired glucose tolerance. The underlying mechanism for this effect might be that acarbose increases the fermentation of insoluble fibers in the colon [25]. Similarly, a clinical study showed that butyrate production is significantly increased during acarbose treatment, whereas the production of acetate and propionate is significantly decreased [26].

The beneficial effects of acarbose on diabetes mellitus, such as reduction of inflammatory cytokines and regulation of SCFA levels, can be related to the alteration of intestinal microbiota. However, whether there is an association between the cardiovascular protective action of acarbose and gut microbiota is not yet clear.

## 4. Glucagon-Like Peptide-1 (GLP-1) and Gut Microbiota

Endocrine peptides play an important role in the crosstalk between gut microbiota and host metabolism. Everard and Cani [60] reviewed the effects of certain dietary fibers on the gut microbiota and host homeostasis and on the secretion of enteroendocrine peptides. As discussed earlier in this review, gut microbiota ferments nondigestible polysaccharides in the colon producing SCFAs and thus influences host glucose and energy homeostasis. It has been elucidated that SCFAs activate GPR41 and GPR43 and thus regulate the secretion of enteroendocrine peptides [61–63]. Known as a gut hormone, GLP-1 is involved in glucose metabolism, appetite regulation, and gastric emptying. The mechanism by which gut microbiota could accelerate the GI motility was mainly attributed to the suppression of GLP-1 receptor expression in the GIT [64]. Recently, several studies have proven the significant glucose-lowering effect of GLP-1-based therapies; GLP-1 receptor agonists like exenatide and liraglutide could promote insulin secretion, suppress glucagon levels, slow gastric emptying, accelerate satiety, induce weight loss, and lower the risk of hypoglycemia [29]. Grasset et al. [65] demonstrated that GLP-1 sensitivity was modulated by gut microbiota through nitric oxide-dependent mechanism in the enteric nervous system. Their results indicated that new hypoglycemic agents based on GLP-1 should focus on the research of patients' intestinal microbiota.

Currently, the association between gut microbiota and intestinal peptides involved in energy and glucose homeostasis has been well reported. However, the relationship between GLP-1 receptor agonists and gut microbiota is not yet clear; further clinical trials are needed to understand this relationship.

## 5. Other Glucose-Lowering Agents and Gut Microbiota

Pioglitazone is a thiazolidinedione that can reduce insulin resistance by binding to peroxisome proliferator-activated receptor *γ* (PPAR-*γ*) nuclear receptor [29]. Previous experimental studies identified the difference in gut microbial structure between KKAy mice and C57BL/6J mice by showing a higher abundance of *Bacteroides* spp. in the intestine of KKAy mice [66]. Pioglitazone was shown to improve gut microbial structure of KKAy mice, but it decreased microbial diversity [66]. The traditional Chinese medicine *Danshensu Bingpian Zhi* (DBZ), a PPAR-*γ* partial agonist, not only improved the phenotypes of metabolic syndrome but also increased the Bacteroidetes/Firmicutes ratio, resulting in an increase in *Akkermansia muciniphila* and a decrease in *Helicobacter marmotae* in HFD-fed mice [67]. Sitagliptin, a dipeptidyl peptidase-4 (DPP-4) inhibitor, was also found to improve gut microbial structure. The underlying mechanism is not clear; however, it can be mediated by relieving intestinal wall edema, alleviating intestinal inflammation, and maintaining the intestinal mucosal barrier integrity [13]. Vildagliptin, another DPP-4 inhibitor, was found to significantly reduce microbiota diversity in diabetic rats and to normalize the Bacteroidetes/Firmicutes ratio [68]. Sodium/glucose cotransporter (SGLT) is a novel therapeutic target in T2DM. LX4211, a dual inhibitor of SGLT1 and SGLT2, could reduce glucose absorption by inhibiting SGLT1 and stimulate the release of GLP-1 and peptide YY. These effects may be mediated by SCFAs produced by cecal fermentation of unabsorbed glucose [69]. Although no definite evidence indicates that SGLT inhibitor has direct effects on gut microbiota to improve glycemic control, it should be noted that SGLT inhibition exerts positive effects on metabolic diseases [70].

Other glucose-lowering agents such as cathelicidin antimicrobial peptide [71], phlorizin [72], and transglucosidase [73] were also proven to have beneficial modulatory effects on gut microbiota composition, glucose homeostasis, insulin resistance, and *β* cell function.

Taken together, these studies are of great significance for understanding the role of gut microbiota as novel therapeutic targets in diabetes mellitus. However, further studies are still needed to confirm the hypothesis.

## 6. Conclusion

In conclusion, the alteration of gut microbiota is a pivotal process in the pathogenesis and progression of metabolic diseases. The association between glucose-lowering agents and gut microbiota is not yet fully understood. Understanding the effects of glucose-lowering agents on gut microbiota is of great importance to further understand the etiopathogenesis, diagnosis, treatment, adverse effects, and prognosis of metabolic diseases.

## Figures and Tables

**Table 1:** Glucose-lowering agents and associated gut microbiota alterations.

Glucose-lowering agent		Gut microbiota alteration		Research subjects
		Increased abundance	Decreased abundance	
Biguanides	Metformin	*Escherichia* [1, 12, 24] *Shigella* [12] *Klebsiella* [12] *Salmonella* [12] *Adlercreutzia* [30] *Clostridium cocleatum* [43] *Akkermansia muciniphila* [24, 43, 44] *Bifidobacterium adolescentis* [24]	*Intestinibacter* [1, 24] *Clostridium* [12] *Eubacterium* [12]	T2DM patients [1, 24, 30] Old women with T2DM [12] HFD-fed mice [43, 44]

*α*-Glucosidase inhibitors	Acarbose	*Bifidobacterium longum* [23, 55] *Lactobacillus gasseri* [23] *Lactobacillus* [58] *Dialister* [58]	*Bacteroides plebeius* [23] *Bacteroides dorei/vulgatus* [23] *Clostridium bolteae* [23] *Butyricicoccus* [58] *Phascolarctobacterium* [58] *Ruminococcus* [58]	T2DM patients [23, 55] Prediabetic patients [58]

PPAR-*γ* partial agonist (Chinese medicine)	Danshensu Bingpian Zhi	*Akkermansia muciniphila* [67]	*Helicobacter marmotae* [67]	HFD-fed mice [67]

DPP-4 inhibitor	Vildagliptin	*Bacteroidetes* [68]	*Prevotellaceae* [68] *Ruminococcaceae* [68]	HFD/STZ SD rats [68]
	Sitagliptin	*Roseburia* [13] *Bifdobacterium* [13]	*Blautia* [13]	HF/HC-STZ SD rats [13]

T2DM: type 2 diabetes mellitus; HFD: high-fat diet; PPAR-*γ*: peroxisome proliferator-activated receptor *γ*; DPP-4: dipeptidyl peptidase-4; STZ: streptozotocin; HF/HC: high fat/high carbohydrate; SD rats: Sprague Dawley rats.
